# Brain Gene Co-Expression Network Analysis Identifies 22q13 Region Genes Associated with Autism, Intellectual Disability, Seizures, Language Impairment, and Hypotonia

**DOI:** 10.3390/genes14111998

**Published:** 2023-10-26

**Authors:** Snehal Shah, Sara M. Sarasua, Luigi Boccuto, Brian C. Dean, Liangjiang Wang

**Affiliations:** 1 Healthcare Genetics and Genomics, School of Nursing, Clemson University, Clemson, SC 29634, USA; snehals@clemson.edu (S.S.); lboccut@clemson.edu (L.B.); 2 Department of Genetics and Biochemistry, Clemson University, Clemson, SC 29634, USA; 3 School of Computing, Clemson University, Clemson, SC 29634, USA; 4 Center for Human Genetics, Clemson University, Greenwood, SC 29646, USA

**Keywords:** 22q13 deletion, Phelan–McDermid syndrome, neurological phenotypes, WGCNA, candidate gene selection

## Abstract

Phelan–McDermid syndrome (PMS) is a rare genetic neurodevelopmental disorder caused by 22q13 region deletions or *SHANK3* gene variants. Deletions vary in size and can affect other genes in addition to *SHANK3*. PMS is characterized by autism spectrum disorder (ASD), intellectual disability (ID), developmental delays, seizures, speech delay, hypotonia, and minor dysmorphic features. It is challenging to determine individual gene contributions due to variability in deletion sizes and clinical features. We implemented a genomic data mining approach for identifying and prioritizing the candidate genes in the 22q13 region for five phenotypes: ASD, ID, seizures, language impairment, and hypotonia. Weighted gene co-expression networks were constructed using the BrainSpan transcriptome dataset of a human brain. Bioinformatic analyses of the co-expression modules allowed us to select specific candidate genes, including *EP300*, *TCF20*, *RBX1*, *XPNPEP3*, *PMM1*, *SCO2*, *BRD1*, and *SHANK3*, for the common neurological phenotypes of PMS. The findings help understand the disease mechanisms and may provide novel therapeutic targets for the precise treatment of PMS.

## 1. Introduction

Phelan–McDermid syndrome (PMS), or 22q13 deletion syndrome, is a rare genetic disorder characterized by autistic traits, varying degrees of intellectual disability (ID), seizures, global delay in speech, severe neonatal hypotonia, minor dysmorphic features, sleep disturbances, gastrointestinal problems, and kidney disorders [1,2]. The syndrome is caused by deletion or disruption in the 22q13 region or pathogenic variants in the *SHANK3* gene [1,3,4]. Deletions vary widely in size from approximately <50 kb to >9 Mb and can include other genes in addition to *SHANK3* [1,5]. Two PMS subgroups have recently been contemplated, based on the involvement of the *SHANK3* gene: PMS-*SHANK3*-related and PMS-*SHANK3*-unrelated [6]. Identifying the specific genes contributing to the different clinical features presented by individuals with PMS has been challenging, particularly due to the generally small sample sizes and lack of investigation [7,8]. Thus, the specific roles in PMS of the vast majority of the 226 protein-coding and non-coding genes in the 22q13.2–13.33 region are largely unknown [9].

To date, several techniques have been used to identify the genes in the 22q13.2–q13.33 region that contribute to the PMS phenotypes along with *SHANK3.* Sarasua [10] used segmental association analysis to identify deletion regions and genes associated with the clinical features. Tabet [11,12] performed a sliding window and hierarchical clustering analysis based on sex, speech, autism spectrum (ASD), seizures, and the size of the deletion along with gene mapping and CNV analysis. Samogy-Costa [13] examined the genotype–phenotype correlation among PMS individuals of diverse ethnicities and the association between genetic altercations in the 22q13 comorbidities using hierarchical clustering. Levy [14] examined the prevalence of clinical features for those with *SHANK3* variants, small deletions including *SHANK3*, and larger deletions. Mitz [8] describes a framework to assess multiple genes near *SHANK3* based on the prevalence of gene loss and predicted loss pathogenicity along with the functional discovery of genes. Ziats [9] examined the normal brain expression levels of 65 protein-coding genes to assess the effects of the developmental stage and brain region for *SHANK3* and the four most highly expressed genes in the brain (*SULT4A1*, *ATXN10*, *MLC1*, and *MAPK8IP2*). Despite the use of various analytical methods to examine the molecular heterogeneity of deletions in the 22q13 region, the variability of clinical features, and the small pool of patients (rare disorder), the specific genetic determinants of PMS have remained unclear. A phenotype-driven approach based on what is known about the genetics of neurobehavioral disorders is needed to identify specific genes related to neurological phenotypes associated with PMS. In this study, we aimed to identify and prioritize the 22q13 candidate genes associated with specific neurological phenotypes and functionally annotate these genes to understand their underlying biological processes.

Weighted Gene Co-expression Network Analysis (WGCNA) [15] was used to identify potential candidate genes that might contribute to the main neurological phenotypes of ASD, ID, seizures, language impairment, and hypotonia in PMS. We performed WGCNA [15] using human brain developmental transcriptomic RNAseq data [16] that could help in identifying groups of genes that tend to be co-regulated and share underlying biological processes to understand the molecular heterogeneity of this complex disorder. We hypothesized that, using this technique to identify 22q13 genes contributing to specific PMS phenotypes, we could identify a list of candidate genes, functionally annotate them to understand their biological processes, and provide new insights into the disease mechanisms of PMS.

## 2. Materials and Methods

### 2.1. Strategy

**Figure 1:**
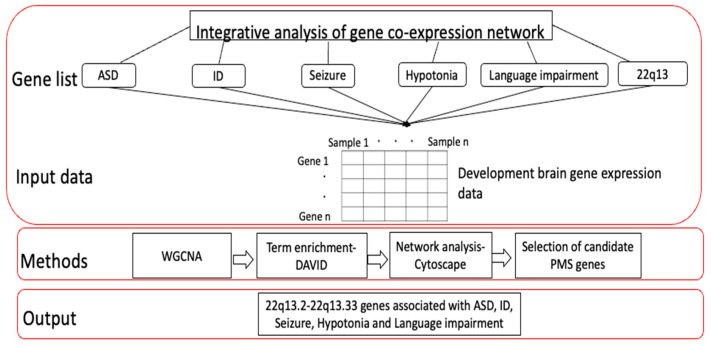
Overview of functional annotation of 22q13 genes using WGCNA. First, curated lists of disease-associated genes for each phenotype and for the list of genes in the 22q13 region were compiled. Then, gene expression data for these genes were extracted from human developmental brain gene expression data. Second, WGCNA was used to identify co-expression modules enriched with 22q13 genes. Third, enriched modules were functionally annotated. Lastly, we identified and prioritized the candidate genes for PMS in the 22q13 region.

The WGCNA package [15] is built in R and has tools for network construction, module detection, gene selection, calculations of topological properties, data simulation, visualization, and interfacing with external software such as the Database for Annotation, Visualization, and Integrated Discovery (version v2023q3) [17] and the Cytoscape (version 3.10.0) visualization tool [18]. WGCNA has been used in previous studies [19,20,21,22,23,24] for identifying and functionally annotating genes associated with neurodevelopmental disorders, interpreting convergent molecular pathways, and visualizing the correlations among genes having similar biological pathways based on the Pearson’s correlation and the degree of connectivity between genes [15]. As shown in Figure 1, WGCNA was used in this study to identify modules of highly co-expressed genes with similar biological processes and pathways based on the expression values of genes in the developing brain [16]. Thus, by performing gene co-expression network analysis, we aimed to identify 22q13 genes sharing similar expression patterns as known phenotype genes by using a principle called “guilt by association”. After identifying modules with 22q13 genes highly correlated to the known phenotypes, we functionally annotate the modules to identify the biological processes and functions that the genes in the 22q13 region might play using DAVID analysis. Finally, the 22q13 genes highly correlated to ASD, ID, seizures, hypotonia, and language impairment would be identified based on the intramodular connectivity (distance between two genes or nodes) using the Cytoscape visualization tool.

### 2.2. Genes Located on 22q13.2–q13.33

The genes in the 22q13.2–q13.33 region were compiled from the UCSC Genome Browser “https://genome.ucsc.edu/goldenPath/releaseLog.html#latest (accessed on 29 December 2020)” [25] for the assembly GRCh37/hg18. This 10.30-Mb region encompasses the deletion regions observed in PMS. A total of 239 protein-coding and non-coding genes (lncRNAs and miRNAs) were extracted from the database. Out of the 239 genes, 207 genes were present in the BrainSpan dataset [16]. A total of 139 genes were included in the final list after filtering genes with no or little expression (expression levels below 0.3 RPKM). A description of the final 139 genes used in the study is shown in Table S1.

### 2.3. Genes Associated with Neurological Phenotypes

For this study, we selected five neurological phenotypes of interest and identified the genes known to be associated with each of them. The ASD risk genes were downloaded from Simons Foundation Autism Research Initiative (SFARI) human gene autism database “https://gene.sfari.org/database/human-gene/ (accessed on 29 December 2020)” [26]. The genes were filtered based on the score criteria provided in the database. The genes falling in the S, 1, and 2 categories (S—syndromic, 1—high confidence, and 2—strong candidate) were selected for the analysis. The genes for ID were downloaded from IDGenetics: a comprehensive database of genes causing ID “https://www.ccgenomics.cn/IDGenetics/ (accessed on 29 December 2020)” [27]. Seizure genes were compiled from a review paper on epilepsy-associated genes [28]. Genes for hypotonia were extracted from NCBI Genes “http://www.ncbi.nlm.nih.gov/gene (accessed on 29 December 2020)” [29] using the search terms: “flappy baby syndrome” and “hypotonia”. The used filters were [species—*Homo sapiens*], [categories—protein coding genes], and [sequence content—Ensembl]. Similarly, the search terms used for language impairment were “speech”, “speech delay”, and “language impairment”, with the same filters of category and sequence content used for hypotonia. The lists of genes compiled for the five neurological phenotypes are shown in Table S1.

### 2.4. BrainSpan Gene Expression Data

The BrainSpan dataset was used in this study “https://www.brainspan.org/ (accessed on 29 December 2020)” [16]. BrainSpan is a developmental transcriptome dataset for the human brain, reported in units of reads per kilobase per million (RPKM) and mapped to genes as annotated using the GENCODE consortium version 10 “https://www.gencodegenes.org/human/releases.html (accessed on 29 December 2020)” [30]. The dataset consists of 524 cadaver samples spanning a developmental period of 8 weeks post-conception to 40 years of age and from up to 16 brain structures. The brain samples were from donors who were neurologically and developmentally normal. Expression was examined for the 2116 genes compiled, as described above, to represent the genes known to be associated with at least one neurological phenotype of interest or to be located on 22q13.2–13.33. If the cumulative gene expression of a gene across 524 samples summed to 0 RPKM (no expression), or if the genes were absent in the BrainSpan repository, they were removed from the dataset. The dataset was *log_2_*(*RPKM* + 1) transformed. Next, the descriptive statistics (mean, median, mode, and standard deviation) of all sample features for each gene were calculated using the dplyr library in R (version 3.6.2). Genes with no or little expression (mean expression < 0.3 RPKM) were filtered out from the dataset. The final dataset comprised a total of 2216 genes (ASD—941, ID—764, seizures—799, hypotonia—248, language impairment—69, and 22q13.2–q13.33 region—139) (Table S1).

### 2.5. Weighted Gene Co-Expression Network Analysis (WGCNA)

The WGCNA package was used to construct gene co-expression networks. The networks were constructed using signed correlation where the correlation was between 1 and 0. The correlation values were stored in a similarity matrix, which is defined between the gene expression profiles, i.e.,

(1) $$n\times n\;similarity\;matrix\;S=[S_{ij}]$$

(2) $$S_{ij}=|cor(x_{i},x_{j})|$$

The similarity matrix is then transformed into an adjacency matrix A = [aij], which encodes the connection strength between the nodes [31].

(3) $$a_{ij}=S_{ij}\beta$$

The adjacency matrix is built using a soft threshold *β* based on a correlation matrix of approximate scale-free topology. In scale-free topology, the degree of distribution follows a power law (the relative change in one quantity results in an exponential change in another). In this study, we identified the power *β* to be 9 by plotting the R^2^ against a soft threshold. The smallest integer value must be selected for a soft threshold such that the resulting network satisfies the approximate scale-free topology (Figure S1). The adjacency matrix is then transformed into a Topological Overlap Matrix (TOM) and the dissimilarity was calculated. The calculated dissimilarity values were used for hierarchical clustering. Hierarchical clustering was conducted using “hclust” based upon the dissimilarity matrix (Figure S2). This process of clustering is utilized for the detection of outliers if any in the samples. Finally, each module holds the expression values compressed into a single value vector called the eigengene vector, and each module eigengene represents the first principal component analysis of the module expression. Modules with highly correlated eigengenes (ME > 0.8) were merged with an average height of 0.25. The size of the module formed was manually set to attain modules with large sizes as fewer samples might bias the results by one or more gene replicates or the module eigengene may not have any meaningful biological insights. To identify modules enriched for 22q13 genes and neurological phenotypes, we performed Fisher’s exact test. The *p*-values were *−*log_10_(*p*-value) transformed for a better visualization of the enriched modules. Using the ggplot package in R, we constructed a heatmap which shows the modules enriched with 22q13 genes and neurological phenotypes.

### 2.6. Functional Term Enrichment Analysis of Co-Expression Modules

The main goal of performing WGCNA in this study was to find the biological and/or clinical significance of the genes on 22q13 associated with the phenotypes of interest. Thus, after identifying the co-expression modules, gene enrichment analysis was performed to understand the biological functions of the genes in the 22q13.2–q13.33 region and investigate how these genes may play important roles in the pathogenesis of PMS. The annotation of the module genes was performed using DAVID “https://david.ncifcrf.gov/tools.jsp (accessed on 20 October 2022)” [17,32], where the enriched biological processes, molecular functions, and cellular components of the genes in a module can be analyzed.

### 2.7. Prioritization of the Candidate Genes

Network visualization can serve as a valuable tool in the identification of hub genes, which are genes that display high connectivity in a co-expression module. Mutations in hub genes often have notable impacts on the functionality of the genes to which they are connected. To identify hub genes in this study, we constructed weighted gene co-expression networks by applying a soft threshold and then created network plots using Cytoscape version 3.10.0. The prioritization of candidate genes was carried out based on their degree of connectivity, as well as the module membership scores of genes located on 22q13.2–q13.33. Additionally, we incorporated the haploinsufficiency score (pLi > 0.9) obtained from the Genome Aggregation Database (gnomAD 2.1.1) “https://www.nature.com/articles/s41586-020-2308-7 (accessed on 20 October 2022)” [33], as PMS is attributed to the haploinsufficiency of genes on 22q13 or on the pathogenic variants of *SHANK3*.

## 3. Results

### 3.1. Identification of Modules Enriched with 22q13 Genes

**Figure 2:**
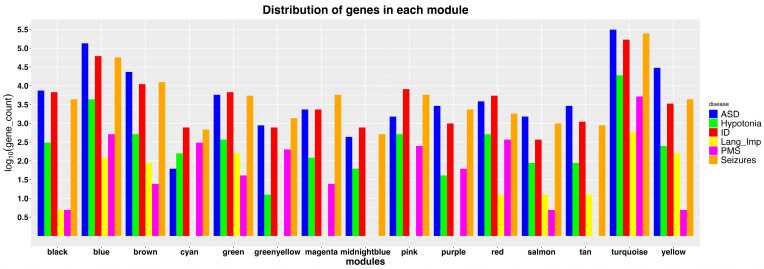
Distributions of 22q13 and neurological phenotype genes in modules after WGCNA. Each module represented by a distinct color is indicated on the *X*-axis. The *Y*-axis shows the log_10_-transformed count of genes in each module. The gene counts for specific neurological phenotypes are indicated by color bars: blue for ASD, green for hypotonia, red for ID, yellow for language impairment (Lang_Imp), pink for genes in the PMS region (22q13.2–22q13.33), and brown for seizures.

Using WGCNA, 16 co-expression modules were identified from a set of 2116 genes. The assumption is that co-expressed genes share similar functions and/or regulatory controls. Therefore, a module that displays enrichment for both ASD genes and the genes in the 22q13 region is considered to show an association of ASD and 22q13 genes. The distributions of the genes for each of the five phenotypes and genes in the 22q13 region (PMS group) in individual modules are shown in Figure 2.

**Figure 3:**
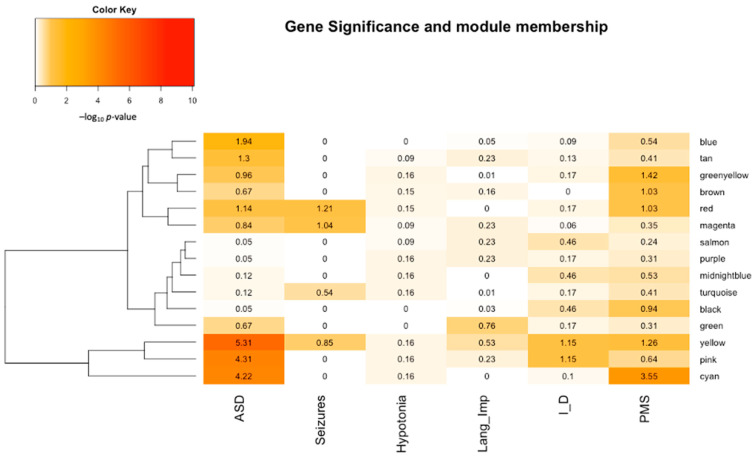
Identification of modules enriched for PMS and neurological phenotypes. The figure depicts the results from the Fisher’s exact tests conducted to identify modules enriched for PMS and other neurological phenotypes. The −log_10_ *p-*values of the correlation between a phenotype and modules are shown. A color key is provided, where darker colors indicate higher enrichment levels.

After clustering the genes based on co-expression into modules, the next step was to identify modules enriched with 22q13 genes. Using Fisher exact test, we identified five modules enriched with 22q13 genes, including the brown, cyan, green-yellow, red, and yellow modules (Figure 3). These five modules also show relatively high gene counts for ASD (Figure 2), while the green, magenta, pink, salmon, and tan modules appear to be more enriched for the other neurological phenotypes (Figure 3).

### 3.2. ASD-Associated Candidate Genes in the 22q13.2–q13.33 Region

To further understand the biological significance of genes in the modules of interest, we conducted functional term enrichment analysis using the Database for Annotation, Visualization, and Integrated Discovery (DAVID). The objective of the analysis was to annotate the gene list in the specific module of interest. ASD’s etiology is heterogenous because of various genetic and environmental factors. In the top three modules enriched for ASD, including cyan, pink, and yellow (Figure 3), we identified biological functions such as histone acetylation, brain development, cilium assembly, the semaphorin–plexin signaling pathway, and the regulation of transcripts from RNA polymerase II. Aberrations of epigenetic modifications such as histone acetylation may cause chromatin remodeling and a change in gene transcription. The changes in DNA can influence how genes related to synaptic functions, neuronal excitability, and immune responses are expressed, and these factors are often linked to the cause of ASD. Regulation of transcripts from RNA polymerase II plays a central role in transcribing mRNA. Dysregulation potentially influences transcription and alternative splicing, which are commonly associated with ASD. The semaphorin–plexin signaling pathway is an essential cellular communication mechanism that regulates various processes during development such as axon guidance and cell migration. Dysregulation in the pathway may lead to developmental abnormalities as seen in ASD. Aberrations in cilium assembly may cause ciliopathies which affect various parts of the body including the brain. Cilium assembly has not been directly linked to ASD; however, neurodevelopmental disorders are complexed and multifaceted.

Following the functional annotation of modules of interest, we identified hub genes that shared a strong correlation with known ASD risk genes. The selected hub genes in the 22q13 region associated with ASD are *TCF20*, *EP300*, *RBX1*, *C22orf32*, *XPNPEP3*, and *SHANK3* (Table 1 and Table S1).

### 3.3. ID-Associated Candidate Genes in the 22q13.2–q13.33 Region

Like ASD, ID is also a neurodevelopmental disorder with heterogenous etiologies, making it difficult for genetic and clinical diagnosis. The yellow and pink modules are enriched for ID genes. The biological processes enriched in these modules are nervous system development, nervous system myelination, and ciliary basal body docking. Dysregulation associated with nervous system development and myelination could lead to ID. Although there is no direct link of ID to dysregulation in ciliary basal body docking, disorders such as Joubert syndrome, Bardet–Biedi syndrome, and Meckel syndrome are ciliopathies that can be associated with ID. The candidate genes in the 22q13 region, including *PMM1*, *LMF2*, *C22orf40*, *CHKB*, and *SCO2* (Table 1), show a strong expression correlation to the known ID genes. Since co-expressed genes can share similar biological and/or regulatory functions, we selected these five 22q13 genes as the candidates associated with ID.

### 3.4. Seizure-Associated Candidate Genes in the 22q13.2–q13.33 Region

The modules enriched for seizures are red, magenta, turquoise, and yellow (Figure 3). The functional terms enriched in these modules include the regulation of ion transmembrane transport, nervous system development, axon guidance, neuron migration, the semaphorin–plexin signaling pathway, brain development, and chemical synaptic transmission. Seizures are commonly observed in patients with ASD and ID, and thus, some of these biological processes such as brain development, the semaphorin–plexin signaling pathway, and nervous system development are also linked to ASD and ID. The other terms, such as ion transmembrane transport, axon guidance, neuron migration, and chemical synaptic transmission, play critical roles in the development of the brain. Aberrations in these processes may cause abnormal neural circuits, abnormal electrical activities, and imbalances in the neurotransmitter systems, which can result in the manifestation of seizures. The hub genes identified in the modules enriched for seizures are *SREBF2*, *BRD1*, *RANGAP1*, *PPP6R2*, *SBF1*, and *SHANK3* (Table 1).

### 3.5. Candidate Genes Associated with Hypotonia and Language Impairment

Two modules, green and yellow, are enriched for the genes associated with language impairment (Figure 3). The green module has co-enrichment for ASD whereas the yellow module is also enriched for ID. The enriched functional terms for the yellow module include homophilic cell adhesion via plasma membrane adhesion molecules and nervous system development. Language impairment, ASD, and ID may share some common underlying causes in genetic factors, brain abnormalities, and environmental factors; therefore, it is not unexpected to see some modules co-enriched for these phenotypes. The candidate 22q13 genes selected for language impairment are *NUP50*, *TUBGCP6*, *ST13*, *ARSA*, and *CPT1B* (Table 1).

The functional analysis did not identify any modules enriched for hypotonia. Thus, our approach could not be used to select candidate genes in the 22q13 region associated with hypotonia. However, there is strong clinical and experimental evidence suggesting the deletion of *SHANK3* in patients with hypotonia [2,34,35,36]. The possible reason for not being able to find a correlation between *SHANK3* and hypotonia could be because gene co-expression analysis may overlook certain features of the complexity of gene–gene interactions.

### 3.6. Network Analysis to Reveal Candidate Gene Interactions

**Figure 4:**
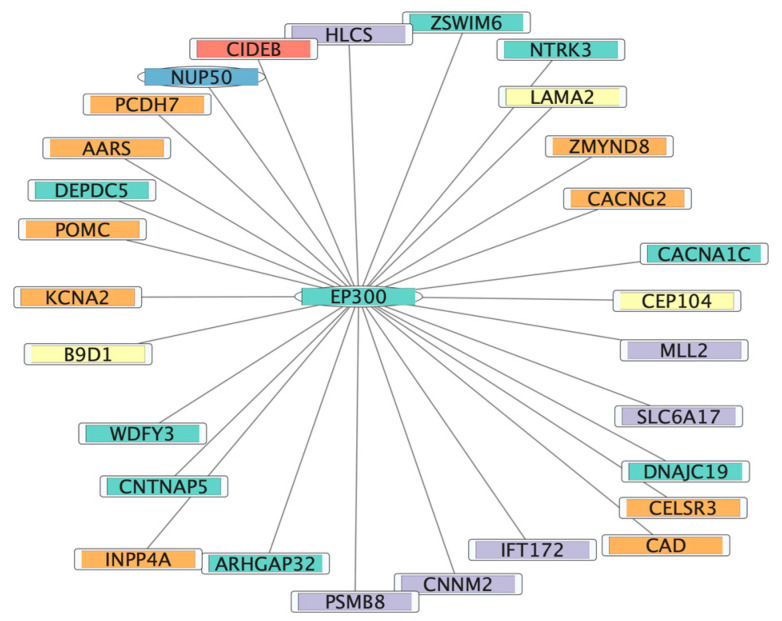
Network visualization to reveal *EP300* gene interactions in the co-expression module using Cytoscape. The figure presents the network topology of *EP300* and neurological phenotypes in the cyan module. *EP300* is represented in turquoise along with PMS genes, and the interacting genes are indicated by different colors: ASD—turquoise, ID—purple, hypotonia—yellow, language impairment—red, and seizures—orange. PMS genes (*EP300* and *NUP50*) are shown in the oval shape.

The functional analysis helped annotate the candidate genes in the 22q13 region. To further understand the interactions among the genes in a module of interest, network analysis and visualization could be performed. We generated the networks using Cytoscape, which showed the connections of genes in the module of interest. The connections between the 22q13 gene *EP300* and the neurological phenotypes are shown in Figure 4. *EP300*, a hub gene in the co-expression network, is associated with Rubinstein–Taybi syndrome, which has similar phenotypes as ASD and patients also exhibit seizure episodes. *EP300* regulates transcription activators and may be involved in autism, intellectual disability, and seizures [37]. Network analysis can help reveal the relationships of genes and their roles in complex diseases (Figure S3).

## 4. Discussion

PMS is characterized by a marked variability in its clinical presentation, which, along with a noticeable genetic heterogeneity, poses a significant challenge to the development of efficacious treatment protocols. Characterizing the contributing roles of the genes in the 22q13 region is fundamental to better understanding the pathogenic mechanisms responsible for the clinical signs and symptoms reported in individuals with PMS. Due to the high number of genes affected by 22q13 deletions associated with PMS and the numerous pathways in which they are involved, gene co-expression network analysis can provide a valuable approach to identifying candidate genes whose heterozygous loss may disrupt the pathways related to the PMS phenotypes. Characterizing the roles of these genes in the pathogenesis of PMS and, more generally in the function and regulation of their pathways, can provide novel targets for the development of precise treatment protocols.

WGCNA, a systems biology approach, was implemented to identify and prioritize candidate genes for selected neurological features of PMS using RNA-seq gene expression data generated from the human brain of normal patients. The purpose of the study was to functionally annotate genes in the 22q13.2–aq13.3 region and select candidate genes that show a high level of expression correlation to known disease genes for traits such as ASD, ID, seizures, hypotonia, and language impairment. This work identified 21 high-confidence candidate genes in the 22q13 region, including six genes *(EP300*, *TCF20*, *RBX1*, *C22orf32*, *XPNPEP3*, and *SHANK3*) for ASD, five genes *(PMM1*, *SCO2*, *LMF2*, *C22orf40*, and *CHKB)* for ID, six genes *(SREBF2*, *BRD1*, *RANGAP1*, *PP6R2*, *SBF1*, and *SHANK3)* for seizures, and five genes (*NUP50*, *TUBGCP6*, *ST13*, *ARSA*, and *CPT1B*) for language impairment. This analysis also identified *SHANK3* as a candidate gene for ASD and seizures. The involvement of *SHANK3*, a gene at the terminal end of chromosome 22, as a cause for neurological impact has been known for quite some time [4,38,39,40,41] and serves as a validation of this method in identifying candidate genes. However, several studies have reported interstitial deletions that preserve the terminal end of 22q13, including *SHANK3*, yet they still have the features of PMS [38,39,42] and are therefore considered as PMS*-SHANK3* unrelated [6]. Individuals with larger deletions—and therefore additional gene loss—tend to be more affected [12,13,14,43,44]. Thus, the WGCNA-based approach in this study is useful for identifying additional candidate genes for the main neurological phenotypes of PMS.

Our investigation used the well-established brain developmental transcriptome dataset [16], which consists of RNA-seq data collected from human brain cadaver samples. The same dataset was used by Ziats [9] to explore the temporal and spatial expression profiles across developmental time points. Based on their studies, *SULT4A1*, *ATXN10*, *MLC1*, *MAPK8IP2*, and *SHANK3* were identified as the most highly expressed genes and thus the potential candidates for PMS. In contrast, our approach identified candidate genes that were not solely based on high expression in the brain. Nevertheless, some of the candidate genes identified via our approach such as *BRD1*, *SBF1*, *SCO2*, *TCF20*, and *CHKB* overlap with the previous findings [8,11]. The overlap of candidate genes from previous work and our study which highlights the co-enrichment of candidate genes with known phenotypes sharing similar biological functions leads closer to the search for identifying 22q13 genes that contribute to the pathogenesis of PMS. The top candidate genes that have a strong association with PMS phenotypes along with high pLI scores available from gnomAD (Table 1 and Table S1) are the protein-coding genes *EP300*, *TCF20*, *SBF1*, *BRD1*, and *SHANK3. SHANK3* is the only well-validated contributor to the neurobehavioral phenotypes of PMS [1,3,34].

The co-expression network analysis identified *EP300* as a candidate gene for autism, intellectual disability, seizure, hypotonia, and language impairment (Figure 4). This gene is responsible for causing Rubinstein–Taybi syndrome 2, a disease sharing clinical features with PMS, such as intellectual disability, growth retardation, and behavioral problems, along with facial dysmorphic features [45,46]. EP300 acts as a transcription coactivator and functions as a histone acetyltransferase (HAT) that regulates transcription via chromatin remodeling [47,48]. The p300 transcription coactivator protein can regulate various signal transduction pathways, which are responsible for the changes in the transcription and or translation of genes, post-translation, and conformational changes in the proteins [49]. Thus, EP300 is involved in biological processes such as cell growth, proliferation, and differentiation [3,49]. Although *EP300* is not always deleted among PMS patients, being located approximately 9 Mb from the telomere, it could have positional effects on the phenotypic features because of its molecular functionalities and evidence of causing Rubinstein–Taybi syndrome 2. Similarly, the *TCF20* gene located 8–9 Mb from the telomere has a pLI score of 1 and is responsible for encoding transcriptional coregulation protein transcription factor 20 which is paralogous to the *RAI1* gene. Pathogenic variants of the *RAI1* gene cause ~90% of cases of Potocki–Lupski syndrome [50,51]. The clinical features associated with the Potocki–Lupski syndrome are developmental delay with varied intellectual impairments and behavioral abnormalities. These clinical features are also seen in patients with PMS. Thus, *TCF20* is another strong candidate gene for neurobehavioral issues in PMS. The pLI score of 1 indicates that the loss of one copy of this gene may result in haploinsufficiency. Hence, *EP300* and *TCF20* were selected in this study as two of the top candidate genes for the neurobehavioral PMS phenotypes.

Along with *SHANK3*, two proximal genes—*SBF1* and *BRD1*—have been shown to be associated with seizures, ASD, and ID. *SBF1* is a target gene commonly associated with a neurological disorder, Charcot Marie Tooth disease (CMT) [52], and is highly expressed in the brain region “https://www.proteinatlas.org/ENSG00000100241-SBF1/tissue (accessed on 20 October 2022)”. The clinical evidence of epilepsy in patients with CMT [53,54,55,56] and hypermethylation of *SBF1* in patients with Drug-resistant Temporal Lobe Epilepsy [57] highlights the role of the *SBF1* gene in epilepsy. The other gene identified in our study, *BRD1*, is associated with neuropsychiatric disorders such as schizophrenia and bipolar disorder along with its impact on neurodevelopmental disorders [58,59]. *BRD1* is responsible for regulating brain development and stem cell differentiation along with its domain interacting with histones and DNA. The regulated transcription of *BRD1* and its importance in brain development and functions have been accentuated by various experiments on mouse models [60,61,62,63,64]. The other potential candidate genes identified via our study such as *RBX1*, *XPNPEP3*, *PMM1*, *LMF2*, *SREBF2*, *RANGAP1*, *PPP6R22*, *NUP50*, and *ST13* have shown a high correlation to neurodevelopmental disorders; however, further clinical or experimental evidence is needed to verify their roles in PMS. Moreover, although there might not be sufficient evidence to support a causative role for the individual candidate genes, they collectively may influence the phenotype of PMS. Thus, these candidate genes may be useful for developing a polygenic risk score that considers them together in assessing the causality and disease severity in individuals with PMS.

The WGCNA method has been used to identify potential candidate genes in various studies ranging from neurodevelopmental disorders to cancer [19,20,21,22,23,24,31]. The list of selected candidate genes largely depends on a fixed threshold of expression correlation. The threshold value determines the over or underestimation of co-expressed genes which may result in false positive or negative associations. It also requires well-validated sets of known genes, which are particularly limited for the phenotypes of language impairment and hypotonia. Thus, the candidate genes identified in this study require further validation. However, the WGCNA-based approach has the potential benefit of identifying candidate genes for rare genetic disorders that have a small sample size for clinical phenotypes and may help in narrowing down the search for the genes responsible for PMS. While our study was constrained in its focus on overall gene co-expression patterns, analyzing spatial and temporal co-expression patterns in the developing brain could be the next step toward gaining a deeper understanding of how these candidate genes influence the development of the certain PMS features and contribute to the clinical variability observed in this syndrome.

## 5. Conclusions

In this study, we constructed human brain gene co-expression networks and used them to select candidate genes, particularly *EP300*, *TCF20*, *BRD1*, and *SBF1* in addition to the well-known gene, *SHANK3*, for the common neurological phenotypes of the Phelan–McDermid syndrome. The findings add to the literature on PMS syndrome and may help in solving the mystery of its pathogenesis. The identified candidate genes provide evidence for the relevance of specific genes in the 22q13 region to neurological phenotypes of PMS and may shed light on novel therapeutic targets. Variants in these genes may act in combinations as modifiers and potential synergistic contributors to the phenotype. Moreover, alterations in these genes may be used to develop biomarkers associated with the severity of the clinical presentation, with the chances of developing certain signs or symptoms, or the responsiveness to specific drugs, allowing to tailor more precise protocols from the management and treatment of individuals with PMS.

## Figures and Tables

**Table 1:** Selection of candidate genes in the 22q13 region. The candidate genes were selected based on the degree of connectivity and module eigengene scores > 0.7. The degree of connectivity was calculated using the cytohubba software of Cytoscape. Genes are listed based on the association to the neurological phenotypes. The haploinsufficiency score for each gene is also provided (NA—not available).

Genes in the 22q13 Region	Functional Annotation	Associated Neurological Phenotype	Degree of Connectivity (%)	pLI Score
EP300	Transcriptional regulation, chromatin modification, DNA repair	ASD	48.99	1
TCF20	Regulation of transcription by RNA polymerase II	ASD	48.46	1
RBX1	DNA damage response, negative regulation of Wnt signaling pathway	ASD	45.16	0.95
C22orf32 (SMDT1)	Mitochondrial ion transport	ASD	43.57	0.18
XPNPEP3	Protein Maturation, proteolysis	ASD	43.57	0
PMM1	Metabolic process, Glycosylation	ID	28.53	0
LMF2	-	ID	16.23	0
SCO2	Sensory organ development, Mitochondrial cytochrome C oxidase assembly	ID	15.31	0
C22orf40 (CDPF1)	-	ID	13.35	0
CHKB	Metabolic processes	ID	12.04	0
SREBF2	Signaling pathway, negative regulation of DNA-templated transcription	Seizures	33.54	0.21
BRD1	Regulation of transcription by RNA polymerase II, Regulation of DNA-templated transcription	Seizures	31.91	1
RANGAP1	Activation of GTPase activity, maintenance of protein in nucleus, nuclear export, nuclear transport	Seizures	31.29	0.03
PPP6R2	Regulation of phosphatase activity	Seizures	29.54	0.16
SBF1	Protein dephosphorylation	Seizures	25.53	1
SHANK3	Brain development, brain morphogenesis, Cell junction assembly	ASD, Seizures	17.9	1
NUP50	Nuclear transport, protein localization to nucleus	Language impairment	39.19	0.28
TUBGCP6	Cytoplasmic microtubule organization, spindle assembly	Language impairment	26.09	0
ST13	Post-translational protein folding	Language impairment	14.49	0.66
ARSA	Intracellular lumen	Language impairment	13.04	0
CPT1B	Fatty acid metabolic process	Language impairment	13.04	NA

## Data Availability

Dataset supporting the findings of this study is available online on Genes website.

## Supplementary Materials

The following supporting information can be downloaded at: https://www.mdpi.com/article/10.3390/genes14111998/s1, Figure S1: Optimal soft threshold selection for building scale-free networks; Figure S2: Sample clustering to detect possible outliers in the dataset; Table S1: List of candidate genes in the 22q13 region, prioritized based on degree of connectivity and module eigengenes; Table S2: List of known genes associated with five neurological phenotypes, including autism spectrum disorder (ASD), intellectual disability (ID), seizures, language impairment, and hypotonia; Table S3: List of genes in the 22q13.2–q13.33 region; Table S4: Combined list of genes for the co-expression network analysis.
