# Supplementary material for: Brain Gene Co-Expression Network Analysis Identifies 22q13 Region Genes Associated with Autism, Intellectual Disability, Seizures, Language Impairment, and Hypotonia
Source: Genes (Basel). 2023 Oct 26;14(11):1998. doi: 10.3390/genes14111998 (PMC10671420; doi:10.3390/genes14111998)
Supplement: Supplementary file 1 [file genes-14-01998-s001.zip › Supplementary_Figures_S1_S2_S3.pdf]

# Supporting Information

# Brain gene co-expression network analysis identifies 22q13 region genes associated with autism, intellectual disability, seizures, language impairment and hypotonia

Snehal Shah [1,2], Sara M. Sarasua [1,*], Luigi Boccuto [1], Brian C. Dean [3] and Liangjiang Wang [2,4,*]

[1] Healthcare Genetics Program, School of Nursing, Clemson University, Clemson, SC 29634, USA
[2] Department of Genetics and Biochemistry, Clemson University, Clemson, SC 29634, USA
[3] School of Computing, Clemson University, Clemson, SC 29634, USA
[4] Center for Human Genetics, Clemson University, Greenwood, SC 29646, USA

[*] Correspondence: liangjw@clemson.edu (LW); smsaras@clemson.edu (SMS)

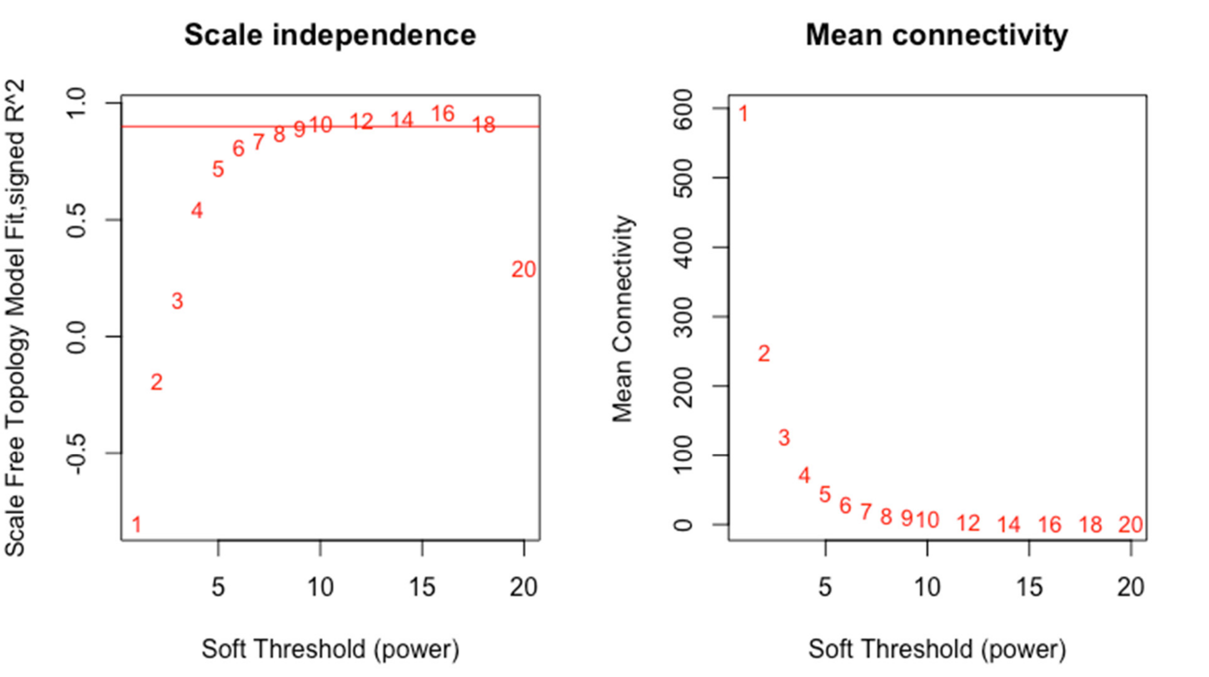


**Figure S1.** Optimal soft threshold selection for building scale-free networks. This helps in identifying densely interconnected modules. In this study, we chose a soft threshold of 9.

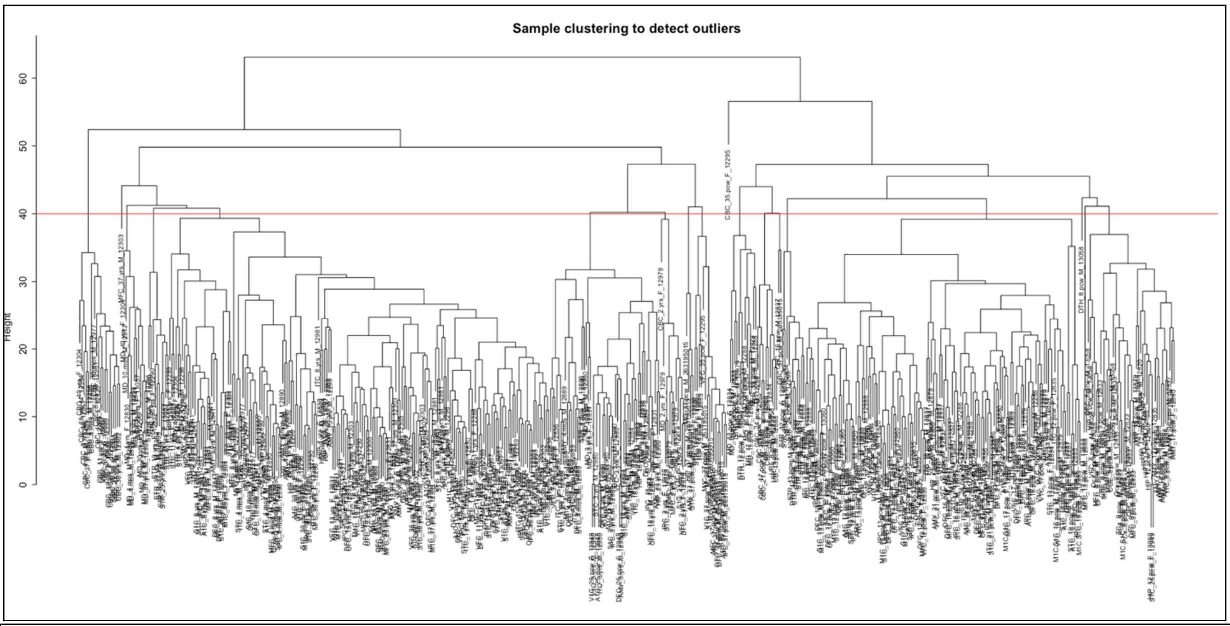


**Figure S2.** Sample clustering to detect possible outliers in the dataset. As a key preprocessing step prior to co-expression network construction, hierarchical clustering is used to detect sample outliers and subsequently remove them from the analysis. By identifying and excluding outliers, the analysis yields more reliable and robust results.

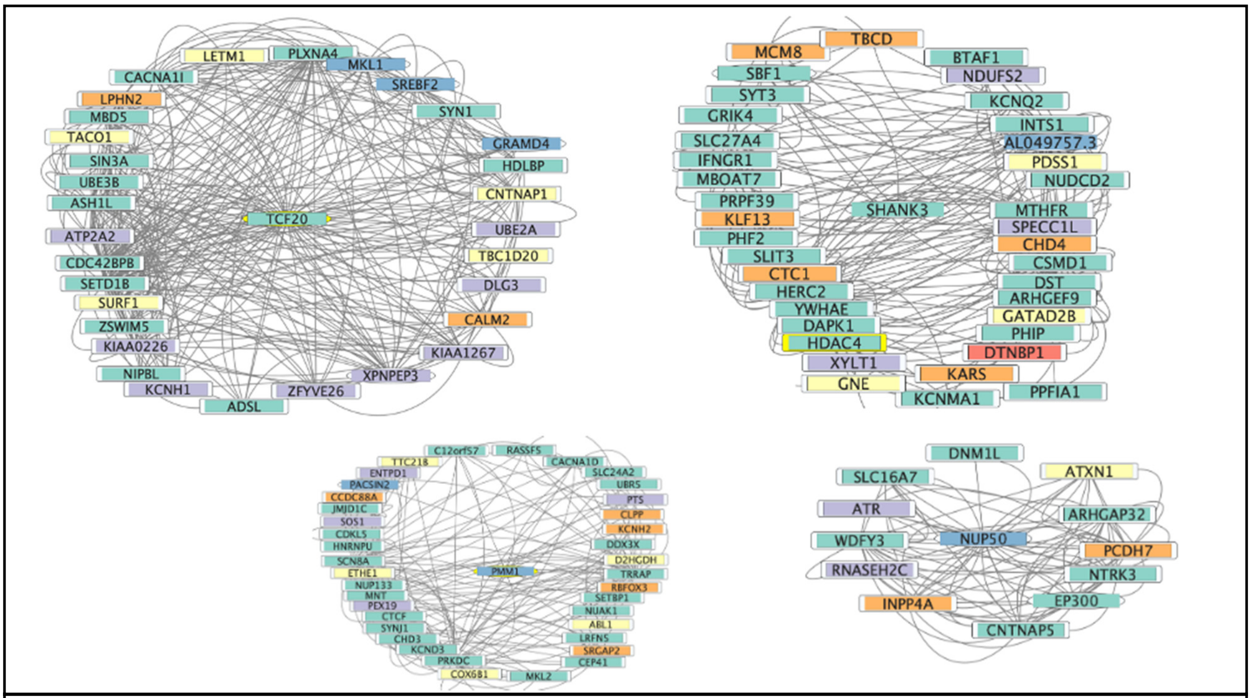


**Figure S3.** Network visualization to show the interactions of hub genes using Cytoscape. The figure illustrates the network topology for *TCF20*, *PMM1*, *SHANK3*, and *NUP50*, all of which are selected candidate genes on 22q13 for the phenotypes, including ASD, ID, seizures and language impairment. The genes are represented in the figure by different colors and shapes, reflecting the specific associated phenotypes: ASD – turquoise, ID – purple, hypotonia – yellow, language impairment – red, seizures – orange. PMS genes are shown in oval shape.
